# Palliative and hegemonic dimensions of conservatism: the mitigating role of institutional trust in shaping attitudes toward migrants and migration policy preferences

**DOI:** 10.3389/fpsyg.2024.1308990

**Published:** 2024-02-15

**Authors:** Matúš Grežo, Magdalena Adamus, Jana Tencerová

**Affiliations:** ^1^Centre of Social and Psychological Sciences, Slovak Academy of Sciences, Bratislava, Slovakia; ^2^Department of Public Economics, Faculty of Economics and Administration, Masaryk University, Brno, Czechia

**Keywords:** conservatism, trust in government, attitudes toward migrants, migration policy preferences, collective narcissism, social dominance orientation, traditionalism, social conservatism

## Abstract

The study explores the links between palliative and hegemonic dimensions of conservatism, attitudes toward migrants and restrictive migration policy preferences. Participants reported on their palliative dimension (social conservatism, traditionalism) and hegemonic dimension (social dominance orientation, collective narcissism) of conservatism, trust in government, attitudes toward migrants, and restrictive migration policy preferences. The results show that both dimensions of conservatism are indirectly linked to more restrictive migration policy preferences through negative attitudes toward migrants. Moreover, the present study indicates that increasing institutional trust may be an effective mechanism mitigating negative attitudes toward migrants for individuals high in the palliative dimension of conservatism.

## Introduction

Migration has become one of the most urgent and most polarising issues in Europe. In 2022 and early 2023, a number of events, such as continuing conflicts in Syria and Sudan, as well as the Russian invasion of Ukraine, forced millions of people to leave their home countries (ICMPD, 2023). Given the hostility that migrants face within new destination countries, there is an urgent need to identify specific psychological and sociocultural factors that drive attitudes toward migrants as key drivers of migration policy preferences and the integration of migrants into new societies.

A large body of literature conducted in WEIRD (Western, Educated, Industrialized, Rich, and Democratic) countries shows that the rejection of and negative attitudes toward migrants and support for more restrictive migration policies are stronger among people who identify as conservatives than among people who identify as liberals (Anderson and Ferguson, 2018; Cowling et al., 2019). This difference tends to be explained by the stronger tendencies of conservatives to be more collectively narcissistic (Lantos and Forgas, 2021), or having higher levels of social dominance orientation (SDO) (Wilson and Sibley, 2013; Golec de Zavala et al., 2019). A recent study conducted by Verkuyten et al. (2022), however, showed that conservatism *per se* can be associated with more positive attitudes toward migrants when collective narcissism (CN) is controlled for. This perhaps surprising finding highlights the importance of delving deeper into diverse motivational roots of conservatives’ attitudes and policy preferences. To understand these motivations, the present study employs the dual-process motivational model (DPM), which allows distinguishing palliative and hegemonic motivational roots of conservatism (Duckitt and Sibley, 2010, 2016). The palliative dimension emerges from feelings of danger and threat and leads to social conservatism and traditionalism. The hegemonic dimension, in turn, is associated with an inflated sense of group-based superiority, supremacist beliefs, and a desire for a dominant intergroup position and leads to CN and SDO.

**Figure 1:**
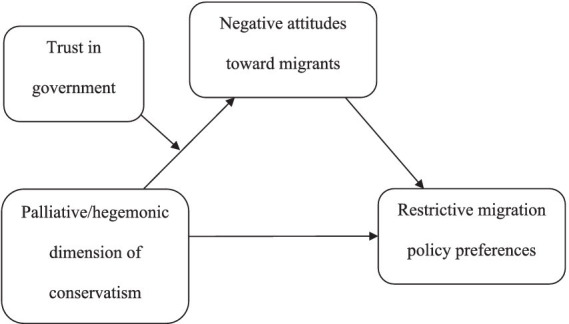
The proposed theoretical model.

The literature indicates that the two dimensions of conservatism, albeit related to each other, could result in support for different policies (Duckitt and Sibley, 2010, 2016). Therefore, combining the recent findings by Verkuyten et al. (2022) with the DPM model (Duckitt and Sibley, 2010, 2016), the aim of the present study is threefold. Firstly, the current study disentangles the role of palliative and hegemonic dimensions of conservatism and investigates how they relate to each other. Secondly, the study investigates how these two dimensions relate to attitudes toward migrants, expressed by fear-based xenophobia and the perceived threat of migrants, and to restrictive migration policy preferences. Finally, recognising the role of trust in shaping relationships with outgroups, we investigate whether trust in government could shield conservative individuals from adopting negative attitudes toward migrants. To achieve these aims, we proposed and tested a moderated mediation model (as shown in Figure 1).

The contribution of this study is twofold. Firstly, the paper contributes to theory by indicating that palliative and hegemonic dimensions of conservatism differ as drivers of attitudes toward migrants and restrictive migration policy preferences. The study examines the links between conservatism, ATM and migration policy preferences in Slovakia — a Central European post-communist country that is at the top of ranking list in terms of negative attitudes toward migrants and preferences for restrictive asylum and refugee policies (Bozogáňová and Piterová, 2020). Secondly, the findings have far-reaching practical consequences indicating that the mechanisms mitigating negative attitudes toward migrants by increasing institutional trust may prove to be effective when conservatism serves the palliative function but futile when it meets hegemonic needs. Thus, with the increasing radicalisation of political views, the portfolio of strategies for attenuating hostility toward migrants may become even more limited.

## Theoretical background

### Negative attitudes toward migrants and conservatism

Over the past decade, immigration has been one of the most pressing issues in Europe. In many European countries, growing dissatisfaction with politicians’ response to the migration crisis has led to a rise in support for populist parties, which often capitalize on the sense of fear and threat posed by immigrants and promote restrictive migration policies (Guzi et al., 2021). Negative attitudes toward migrants and restrictive migration policy preferences have been shown to be particularly strong in Central and Eastern European regions (Schlueter et al., 2013; Heath and Richards, 2016, 2019, 2020; Csanyi and Kucharčík, 2023; Hlatky, 2023). Unsurprisingly, researchers seek to understand the psychological roots of these attitudes in order to potentially reduce negative feelings of fear and threat of immigrants and dampen prejudice and hostility toward them.

The literature points to conservative attitudes as one of the most important predictors of attitudes toward migrants. There is ample evidence that individuals who identify as conservatives are likely to show negative attitudes, prejudice, and hostility toward migrants (Skitka et al., 2002; Inbar et al., 2009; Jost et al., 2009; Luguri et al., 2012; Hainmueller and Hopkins, 2014; Kugler et al., 2014; Brooks et al., 2016; Anderson and Ferguson, 2018; Cowling et al., 2019) and perceive migrants to be a threat to the culture and society (Raijman and Semyonov, 2004). Consequently, conservatism is associated with the exclusion of minorities and migrants and the rejection of their rights (Gorodzeisky and Semyonov, 2009), which translates also into preferences for more restrictive migration policies (Canetti et al., 2016).

The literature proposes two main explanations as to why individuals who identify as conservatives hold negative attitudes toward migrants. The first explanation stems from meta-analytical studies conducted by Jost et al. (2003, 2017), who documented that conservatism is a form of motivated social cognition that is adopted to reduce negative feelings of fear and threat and to avoid uncertainty. Apparently, migrants represent a symbolic threat to conservatives’ social identity and disrupt their need for order, predictability and safety. Moreover, the presence of migrants and refugees increases the diversity of values and worldviews and makes society less homogeneous. To cope with such feelings of fear and threat, individuals with conservative attitudes adopt fear-based, xenophobic attitudes toward migrants (Pazhoohi and Kingstone, 2021), tend to revere to past and adhere to traditional values and stances (Pless and Houtman, 2021), and advocate for preserving social, cultural and moral foundations (De Cristofaro et al., 2019).

The second explanation is that conservatism is positively linked to negative attitudes toward migrants due to its overlap with SDO (Ho et al., 2012; Wilson and Sibley, 2013) and CN (Golec de Zavala et al., 2019; Lantos and Forgas, 2021). Social dominance orientation refers to the individual’s preference for group-based hierarchy and inequality (Ho et al., 2015). Individuals high in SDO believe in and support supremacist beliefs that certain groups are superior to others (Pratto et al., 1994). This, in turn, results in support for policies that maintain and reinforce social hierarchies, such as those based on gender, race, and social class. These beliefs translate into prejudice and negative attitudes toward and the persecution of minorities and migrants (Ho et al., 2015; Anderson J., 2018; Anderson J. R., 2018; Cowling et al., 2019), as well as aggressive intergroup attitudes (Ho et al., 2012), and into petrifying the marginalised status of disadvantaged groups (Sibley et al., 2013).

Collective narcissism, in turn, refers to a supremacist belief in the exceptionality and superiority of one’s own social group. Individuals high in CN tend to have an inflated sense of group-based self-importance and a need for recognition and admiration from others (Golec de Zavala et al., 2019). Like SDO, CN contributes to several disruptive societal phenomena, such as intergroup conflicts, intentions to harm, retaliatory aggression and prejudice, justifying violence, and a preference for military aggression (de Zavala et al., 2009; Golec de Zavala et al., 2013, 2019; Golec de Zavala and Lantos, 2020; Cichocka et al., 2022). Although research in CN has mainly focused on individual characteristics, a recent study by Cichocka et al. (2023) examined country-level characteristics that could explain why some countries display higher levels of collective narcissism than others. The study showed that citizens of less globalized countries exhibit higher CN. However, Slovakia proved to be an “outlier” in this investigation, scoring high on both globalization and CN. The study (Cichocka et al., 2023) does not provide an explanation of this puzzling result. However, we could speculate that the second investigated factor – the sense of victimisation and the belief that the group is disadvantaged compared to others – could prove to be relevant in the Slovak context. Given the historical context of the dissolution of Czechoslovakia, many Slovaks still feel resentful and have a deeply rooted sense of being exploited and betrayed at the time of secession – which, according to the literature, could contribute to their heightened endorsement of CN (e.g., Marchlewska et al., 2018).

### Disentangling palliative and hegemonic dimensions of conservatism

The two described explanations suggest that there exist distinct psychological roots regarding why conservatives may hold negative attitudes toward migrants. These distinct motivational roots are well described by the DPM model proposed by Duckitt and Sibley (2010). The model posits that there exist two distinct motivational bases of prejudice toward outgroups. In particular, negative attitudes toward an outgroup may arise either because the outgroup is seen as dangerous and threatening or because it is seen as a competitor for power or resources. The former motivational basis is associated with rather mild conservative attitudes represented by social conservatism and traditionalism (Duckitt and Sibley, 2010). These attitudes are characterized by the need to maintain social and collective security, stability, and cohesion through obedience to authority, preserving clear rules and traditional values, norms and morality (Duckitt and Bizumic, 2013; Duckitt and Sibley, 2016). Importantly, these needs serve mainly palliative functions. As Sinn and Hayes (2018) argue, those motivations emerge as defensive responses to negative feelings of threat and uncertainty caused by outgroups and offers individuals a sense of order, security and stability (Jost et al., 2008).

In contrast, the latter motivational orientation described by the DPM model is based on the belief that the world is a ruthlessly competitive jungle in which the strongest wins and the weak and unfit lose (Duckitt and Sibley, 2010, 2016). In this worldview, the outgroups are not perceived as a threat, but as competitors in the race for power, social status, or resources. This leads individuals to the desire to dominate over (or even exploit) outgroups – particularly those perceived as inferior – which is reflected in SDO. In this view, SDO serves a hegemonic function, as it arises from dominance-driven motives and beliefs about social hierarchy in which some groups have a superior position (Duckitt and Sibley, 2010, 2016). Such a worldview is associated with deceptive tactics and self-advancement driven by a desire to control and exploit disadvantaged groups or individuals (Duckitt and Sibley, 2010; Sinn and Hayes, 2018).

A recent study by Verkuyten et al. (2022) emphasizes how important it is to distinguish between the palliative and hegemonic dimensions of conservatism, as they can lead to different attitudes toward migrants. By means of three national samples from two European countries, they found that conservatism, represented by self-identification question, was associated with positive attitudes and tolerance toward and support for the rights of migrants and outgroups once controlling for the effect of CN. These findings corroborate the palliative–hegemonic distinction and suggest that those two motivational roots may lead to different attitudes toward migrants. Through SDO and CN, conservatism may be associated with the need to retain or fantasise about a dominant position of one’s own group, feelings of self-importance, and, ultimately, hostility toward outgroups. After excluding this hegemonic dimension, however, conservatism *per se* may provide an adaptive sense of secure belonging, which, in turn, may be associated with more positive attitudes toward migrants and outgroups (Bertin et al., 2022).

Along with the DPM model (Duckitt and Sibley, 2010, 2016), the findings of Verkuyten et al. (2022) have led us to disentangle the palliative dimension of conservatism, represented by social conservatism and traditionalism, from the hegemonic dimension represented by SDO and CN. Although we hypothesize that both palliative (H1) and hegemonic (H2) dimensions of conservatism indirectly relate to restrictive migration policy preferences through negative attitudes toward migrants (Canetti et al., 2016), we expect that (H3) the palliative dimension expresses a weaker indirect relationship with restrictive migration policy preferences than does the hegemonic dimension (Anderson and Ferguson, 2018; Cowling et al., 2019).

### Trust as a potential facilitator of positive attitudes toward migrants

Although potent, conservatism is not the only factor associated with attitudes toward migrants. The present study focuses on trust as a key factor of social capital that has been consistently found to be associated with attitudes toward migrants. In particular, individuals showing high interpersonal trust (Herreros and Criado, 2009; Van der Linden et al., 2017; Mitchell, 2021; Pellegrini et al., 2021) and institutional trust (Husfeldt, 2004, 2006; Halapuu et al., 2013; Economidou et al., 2017; McLaren, 2017; Serrano-Maillo, 2018; Jylhä et al., 2022) are more likely to have positive attitudes toward migrants and refugees, or outgroups in general. Similar to other countries, it was also found in Slovakia that trust is associated with more positive attitudes toward migrants (Bozogáňová and Piterová, 2020; Sedlár, 2023, 2024). Importantly, there is evidence that trust may reduce feelings of threat and, thus, dampen the relationship between political orientation and negative attitudes toward migrants and outgroups. Based on group threat theory, Mitchell (2021) found support for the idea that environments characterised by high distrust make group-based identities more salient and prompt people to believe that outgroups are inferior and intrinsically different, which engenders feelings of threat. In contrast, trusting environments prevent people from thinking that others conspire against them and, thus, they do not perceive outgroups to be threatening. This mechanism could indeed explain the results from Slovakia (as well as from other post-communist countries in the region), which show that the average level of trust in Slovakia is very low (Grežo et al., 2022), while the country is at the top of the EU ladder in terms of negative attitudes.

In the context of our study, a beneficial effect of trust may especially help individuals with conservative attitudes to reduce aversive feelings of fear, threat and uncertainty that they experience (Jost et al., 2003, 2017) and promote feelings of secure belonging (Verkuyten et al., 2022) and cause them to be more open and more tolerant toward migrants. Thus, we hypothesize that (H4) trust in government moderates the relationship between the palliative dimension of conservatism and attitudes toward migrants.

The same process, however, may not work for individuals high in the hegemonic dimension of conservatism, since it stems from the motivational goals of dominance and superiority over others (Duckitt and Sibley, 2010, 2016). Factors such as SDO and CN, representing more extreme or more radical views (Jasko et al., 2020), have been found not to be associated with the desire to reduce feelings of threat and uncertainty (Jost et al., 2007); therefore, the effect of trust on reducing feelings of threat may not be beneficial for individuals high in these factors. As Jost and Napier (2012, p. 91) state, “psychological needs to reduce uncertainty and threat are associated with political conservatism in particular and not ideological extremity in general.” Thus, the hegemonic dimension of conservatism may maintain the relationship with negative attitudes toward migrants across different levels of trust. Therefore, we hypothesize that (H5) the relationship between the hegemonic dimension of conservatism and attitudes toward migrants is not moderated by trust in government.

## Materials and methods

### Participants and procedure

The study was conducted on a Slovak sample in autumn 2021, immediately after the Taliban takeover of Kabul, which triggered a massive migration of Afghans to neighbouring countries as well as Europe. Slovakia — a Central European post-communist country — is not a traditional destination country for migrants, but rather a transit country. Similar to other European Union countries, Slovakia’s migration policy is determined by international treaties (United Nations, Council of Europe, and International Labour Organization) and EU regulations. However, Slovakia has long lacked a coherent immigration policy and currently finds itself at the end of a tail when it comes to immigration rates among European countries (Stojarová, 2019). Political parties and candidates that hold opposite positions towards migration have a considerable support in this country. At the European level, Slovakia is among the most restrictive countries in terms of both integration and immigration (Stojarová, 2019). In 2015, Slovakia led the opposition to the mandatory relocation scheme (quotas) for refugees approved by the EU (Csanyi and Kucharčík, 2023). Unsurprisingly, these facts contribute to maintain the country’s cultural homogeneity and lead Slovak citizens to have negative attitudes toward migrants and favour restrictive asylum and refugee policies (Bozogáňová and Piterová, 2020).

A sample of 600 Slovaks aged 16 to 87 years participated in the survey-based study. The means and standard deviations for the participants’ demographic variables are shown in Table 1. The study was representative of the Slovak population in terms of gender and age distribution. In particular, the sample was gender-balanced (300 women and 300 men), 81.3% were of productive age (aged 15–64) and 18.7% were of post-productive age (aged 65+). However, our sample had a slightly higher level of education than the general Slovak population. We were able to reach only 3.2% of people with primary education, while 56% of participants had a university degree.

**Table 1:** Descriptive statistics and correlation matrix.

Variable	*M*	SD	1.	2.	3.	4.	5.	6.	7.	8.	9.	10.	11.	12.	13.
1. Sex	—	—	—												
2. Age	45.81	16.35	−0.07	—											
3. Education	4	1.26	0.09*	0.20***	—										
4. Religiosity	3.98	2.17	0.05	−0.05	0.04	—									
5. Traditionalism	3.14	0.78	−0.10*	0.16***	<0.01	0.23***	—								
6. Social conservatism	4.16	1.38	−0.09*	0.09*	−0.05	0.21***	0.58***	—							
7. Social dominance orientation	2.37	0.67	−0.09	−0.05	−0.02	0.09*	0.27***	0.22***	—						
8. Collective narcissism	2.73	0.73	−0.04	−0.08	−0.18***	0.12**	0.21***	0.18***	0.19***	—					
9. Trust in government	2.06	0.84	<0.01	−0.05	0.02	0.12**	−0.23***	−0.17***	−0.02	−0.03	—				
10. Fear-based xenophobia	3.28	0.81	<0.01	0.18***	−0.07	0.07	0.48***	0.38***	0.18***	0.33***	−0.29***	—			
11. Perceived threat of migrants	3.04	0.91	−0.06	0.19***	−0.07	0.11**	0.48***	0.37***	0.22***	0.36***	−0.25***	0.84***	—		
12. Migration threats	5.98	1.89	0.01	0.09*	−0.13**	0.10*	0.47***	0.41***	0.14***	0.23***	−0.29***	0.74***	0.73***	—	
13. Migrants’ access	2.34	0.63	0.05	0.12**	−0.06	0.08*	0.38***	0.30***	0.20***	0.23***	−0.18***	0.64***	0.64***	0.64***	—
14. Preferences for restrictive asylum and refugee policies	3.31	0.74	−0.06	0.19***	−0.04	0.02	0.47***	0.37***	0.22***	0.28***	−0.33***	0.70***	0.68***	0.64***	0.64***

Red color indicates negative correlations and blue color indicates positive correlations. The more saturated the color, the stronger correlation. Higher scores for migrants’ access means that participants preferred to restrict the access of migrants to the country, higher score in preferences for restrictive asylum and refugee policies indicates that participants preferred more restrictive migration policies. * *p* < 0.05, ** *p* < 0.01, *** *p* < 0.001.

Participation was anonymous and voluntary and participants could withdraw from the survey at any time. The data was collected via an online survey hosted on Qualtrics. We set three criteria for participation. In particular, we wanted a gender-balanced sample from all Slovak regions and participants had to be Slovak citizens so that they would perceive migrants as outgroups. The data was collected by an online panel research company that provides data collection and market research services for various research and private organizations. The research company used its own panel of respondents and contacted only those individuals who were eligible to participate in the study according to the conditions we set. After reading and signing the informed consent form, participants answered sociodemographic questions on age, sex and education. Thereafter, they reported on the palliative and hegemonic dimensions of conservatism, trust in government, negative attitudes toward migrants, and restrictive migration policy preferences. The complete questionnaire in English along with the dataset are available at the Open Science Framework repository.^1^ The study was approved by the ethical committee of the Centre of Social and Psychological Sciences of the Slovak Academy of Sciences.

### Measures

#### Palliative dimension of conservatism

Based on the DPM model (Duckitt and Sibley, 2010, 2016), the palliative dimension of conservatism was represented by the two distinct but related constructs of social conservatism and traditionalism. *Traditionalism* was assessed using a four-item scale from American National Election Studies (ANES) (2019). The scale includes statements like *“The world is always changing and we should adjust our view of moral behaviour to those changes”* and *“Newer lifestyles are contributing to the breakdown of our society.”* Participants answered on a five-point Likert scale (1 = strongly disagree, 5 = strongly agree). Higher score meant higher traditionalism.

*Social conservatism* was measured using an ideological self-identification question (Conover and Feldman, 1981) that asked participants to rate their orientation as follows: *“On social issues, where would you place yourself from a conservative to liberal-oriented person?”* Participants responded on a seven-point scale (1 = very liberal, 7 = very conservative). Higher score meant that the person was more conservatively oriented.

#### Hegemonic dimension of conservatism

The hegemonic dimension of conservatism was measured using the two scales of social dominance orientation and collective narcissism. The *social dominance orientation scale* (Pratto et al., 2013) was used to measure individual differences in group-based discrimination (e.g., *“Superior groups should dominate inferior groups”*). The scale consists of four items answered on a five-point Likert scale (1 = strongly disagree, 5 = strongly agree). Higher score meant higher social dominance orientation.

Collective narcissism was assessed using the *collective narcissism scale* (Golec de Zavala et al., 2017). The scale consists of five items (e.g., *“My group deserves special treatment”*) answered on a five-point Likert scale (1 = strongly disagree, 5 = strongly agree). Higher score meant higher collective narcissism.

#### Trust in government

An 11-item *trusting beliefs scale* (McKnight et al., 2002) was adapted to measure trust in government. This measure is designed in a way that it allows to modify not only the subject of trust but also the context in which the trustor should be trusted. Thus, we modified the measure to include questions on trust in the Slovak government’s ability to manage the migration crisis efficiently. The scale consists of three subscales: Benevolence (e.g., *“Slovak government is interested in my well-being, not just its own”*), Integrity (e.g., *“Slovak government is sincere and genuine”*) and Competence (e.g., *“In general, Slovak government is very knowledgeable about the migration”*). Participants answered on a five-point Likert scale (1 = strongly disagree, 5 = strongly agree). Higher score meant that person perceived Slovak Government as trustful.

#### Negative attitudes toward migrants

Negative attitudes toward migrants were assessed using three separate measures. Firstly, the *fear-based xenophobia scale* (Van der Veer et al., 2011) is a nine-item scale mapping people’s fear of migration and migrants, which is based on the perception of threat from foreigners (e.g., *“Interacting with immigrants makes me uneasy”*). Participants indicated how threatened they felt on a five-point Likert scale (1 = strongly disagree, 5 = strongly agree). Higher score meant higher fear-based xenophobia.

Secondly, *perceived threat of migrants* (Cottrell and Neuberg, 2005) was used to measure to what extent people perceive migrants to be either a symbolic or a realistic threat to the Slovak Republic. The scale consists of nine items (e.g., *“Immigrants and foreign workers threaten our personal possessions”*) answered on a five-point Likert scale (1 = strongly disagree, 5 = strongly agree). The higher the score, the more people perceived migrants as threat.

Thirdly, *migration threats* was represented by a three-item questionnaire (European Social Survey, 2014). The questions map how people judge migrants in terms of their effects on the national economy, culture, or everyday living *(“Would you say it is generally bad or good for Slovakia’s economy that people come to live here from other countries?”)* The questions are answered on a seven-point scale (e.g., 1 = good for the economy, 7 = bad for the economy). The higher the score, the more people perceived migrants as threat to the Slovak economy, culture, and everyday living.

#### Restrictive migration policy preferences

Restrictive migration policy preferences were assessed using two distinct measures. Firstly, a three-item *migrants’ access* questionnaire (European Social Survey, 2014) assessed participants’ opinions on the extent to which people from other countries should be allowed to live in Slovakia (e.g., *“To what extent do you think Slovakia should allow people of the same race or ethnic group as most Slovak people to come and live here?”*). The questions were answered on a four-point scale (e.g., 1 = allow many to come and live here, 4 = allow none). The higher the score, the more people preferred to restrict the access of migrants to the country.

Secondly, the original six-item *preferences for restrictive asylum and refugee policies scale* was created to assess people’s preferences for what specific asylum and refugee policies a state should follow (e.g., *“Each EU country should make its own decisions on asylum applications within its territory”*). The scale was created based on the work of Jeannet et al. (2021), who identified six core dimensions that characterise the asylum and refugee policies of high-income countries. These dimensions relate to the right of refugees to apply for asylum, the resettlement of already recognised refugees, the return of asylum seekers whose applications for protection have been unsuccessful, family reunification for recognised refugees, the state’s independence regarding their migration policies, and financial solidarity with countries that host refugees. Participants answered on a five-point Likert scale (1 = strongly disagree, 5 = strongly agree). The higher the score, the more people preferred restrictive asylum and refugee policies.

#### Control variables

To control for the effects of sociodemographic characteristics, participants were asked questions on their gender, age, education and religiosity.

## Results

### Descriptive statistics and correlation matrix

Descriptive statistics and a correlation heatmap for the observed study variables are reported in Table 1. As can be seen, we found positive weak to moderate associations between traditionalism, social conservatism, SDO, and CN. In addition, these variables showed positive weak to moderate associations with the variables of negative attitudes toward migrants (fear-based xenophobia, perceived threat of migrants, migration threats) as well as restrictive migration policy preferences (migrants’ access, preferences for restrictive asylum and refugee policies). Finally, the variables of negative attitudes toward migrants and restrictive migration policy preferences showed strong positive intercorrelations.

### Testing the proposed moderated mediation models

Before testing the proposed moderated mediation models, we computed factor scores for the palliative dimension of conservatism (social conservatism + traditionalism), hegemonic dimension of conservatism (SDO + CN), negative attitudes toward migrants (fear-based xenophobia + perceived threat of migrants + migration threats), and restrictive migration policy preferences (migrants’ access + preferences for restrictive asylum and refugee policies) latent constructs by means of a least squares regression method. The factor scores were then used in the analyses of the proposed moderated mediation models.

#### Moderated mediation model with palliative dimension of conservatism

To investigate the moderated mediation model of the palliative dimension of conservatism, a moderated mediation analysis was performed using SPSS PROCESS Macro version 3.4, Model 7 (Hayes, 2013). The model included the palliative dimension of conservatism as a predictor, restrictive migration policy preferences as an outcome variable, negative attitudes toward migrants as a mediator, and trust in government as a moderator. In addition, four covariates (sex, age, education and religiosity) were included in the analysis to statistically remove their potential confounding effects.

The total and direct estimated regression coefficients are displayed in Table 2. As can be seen, negative attitudes toward migrants were positively predicted by the palliative dimension of conservatism, age, and education. In contrast, trust in government and its interaction with the palliative dimension of conservatism negatively predicted attitudes toward migrants. The interaction between the palliative dimension of conservatism and trust in government significantly increased the explained variance of negative attitudes toward migrants: *F*(1, 592) = 16.23; *∆R^2^* = 0.02; *p* < 0.001.

**Table 2:** Total and direct effects in the moderated mediation model with the palliative dimension of conservatism as a predictor.

Variable	*b*	SE	*t*	*p*	95% CI [LL, UL]
Outcome: negative attitudes toward migrants					
*R^2^* = 0.35, *F*(7, 592) = 45.31, *p* < 0.001	–	–	–	–	–
Palliative conservatism	0.77	0.08	9.18	<0.001	[0.60, 0.93]
Trust in government	−0.24	0.04	−5.71	<0.001	[−0.32, −0.16]
Interaction	−0.16	0.04	−4.03	<0.001	[−0.24, −0.08]
Sex	0.10	0.07	1.54	0.12	[−0.03, 0.23]
Age	0.01	<0.01	2.98	<0.01	[0.002, 0.01]
Education	−0.08	0.03	−3.10	<0.01	[−0.14, −0.03]
Religiosity	0.01	0.02	0.77	0.44	[−0.02, 0.04]
Outcome: restrictive migration policy preferences					
*R^2^* = 0.63, *F*(6, 593) = 166.13, *p* < 0.001	–	–	–	–	–
Palliative conservatism	0.09	0.03	3.07	<0.01	[0.03, 0.15]
Negative attitudes toward migrants	0.74	0.03	24.67	<0.001	[0.68, 0.80]
Sex	0.03	0.05	0.66	0.51	[−0.07, 0.13]
Age	<0.01	<0.01	1.10	0.27	[−0.001, 0.004]
Education	0.01	0.02	0.61	0.54	[−0.03, 0.05]
Religiosity	−0.02	0.01	−1.64	0.10	[−0.04, 0.004]

Restrictive migration policy preferences were directly predicted by the palliative dimension of conservatism and negative attitudes toward migrants, with the latter having the strongest positive effect among all observed predictors. Together with covariates, the palliative dimension of conservatism and negative attitudes toward migrants explained 63% of the variance of restrictive migration policy preferences.

**Figure 2:**
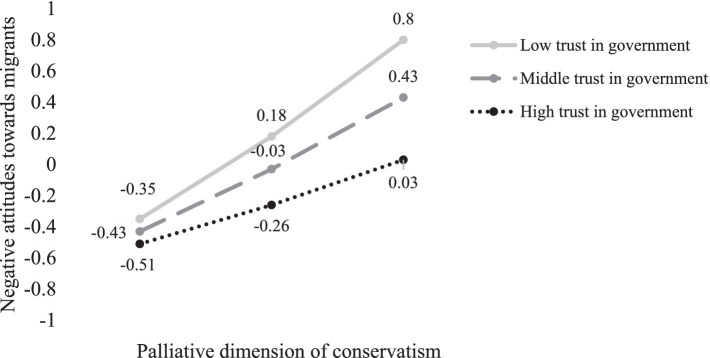
Conditional effects of the palliative dimension of conservatism on negative attitudes toward migrants at different values of trust in government. Figure shows unstandardized regression coefficients.

Conditional effects of the palliative dimension of conservatism on negative attitudes toward migrants at different values of trust in government are presented in Figure 2. In particular, there was a significant positive linear effect of the palliative dimension of conservatism on negative attitudes toward migrants at all levels of trust in government, but this effect was clearly stronger as trust decreased. A 95% bootstrap confidence interval for the moderated mediation index did not include zero (*Index* = −0.12; *BootSE* = 0.04; *95% CI* [−0.19, −0.05]), indicating that the indirect effect of the palliative dimension of conservatism on restrictive migration policy preferences through negative attitudes toward migrants was negatively moderated by trust in government.

#### Moderated mediation model with hegemonic dimension of conservatism

The analogous moderated mediation analysis using PROCESS was utilised to examine the model with the hegemonic dimension of conservatism. The model included the hegemonic dimension of conservatism as a predictor, restrictive migration policy preferences as an outcome variable, negative attitudes toward migrants as a mediator, and trust in government as a moderator. As in the previous model, four covariates (sex, age, education and religiosity) were included in the analysis to control for their potential confounding effects.

The total and direct estimated regression coefficients are displayed in Table 3. We found slightly different results in comparison to the previous model. In particular, negative attitudes toward migrants were positively predicted by the hegemonic dimension of conservatism, but the effect was considerably lower than the effect of the palliative dimension of conservatism in the previous model. In addition, trust in government negatively predicted negative attitudes toward migrants, but its interaction with the hegemonic dimension of conservatism did not significantly predict the attitudes. The interaction between the hegemonic dimension of conservatism and trust in government did not significantly increase the explained variance of negative attitudes toward migrants: *F*(1, 592) = 0.38; *∆R^2^* < 0.001; *p* = 0.54.

**Table 3:** Total and direct effects in the moderated mediation model with hegemonic conservatism as a predictor.

Variable	*b*	SE	*t*	*p*	95% CI [LL, UL]
Outcome: negative attitudes toward migrants					
*R^2^* = 0.25, *F*(7, 592) = 28.75, *p* < 0.001	–	–	–	–	–
Hegemonic conservatism	0.28	0.09	3.22	<0.01	[0.11, 0.45]
Trust in government	−0.34	0.04	−8.04	<0.001	[−0.43, −0.26]
Interaction	0.02	0.04	0.62	0.54	[−0.05, 0.10]
Sex	0.06	0.07	0.87	0.39	[−0.08, 0.20]
Age	0.01	<0.01	5.63	<0.01	[0.01, 0.02]
Education	−0.07	0.03	−2.57	0.01	[−0.13, −0.02]
Religiosity	0.04	0.02	2.83	<0.01	[0.01, 0.08]
Outcome: restrictive migration policy preferences					
*R^2^* = 0.63, *F*(6, 593) = 166.07, *p* < 0.001	–	–	–	–	–
Hegemonic conservatism	0.08	0.03	3.05	<0.01	[0.03, 0.14]
Negative attitudes toward migrants	0.76	0.03	27.62	<0.001	[0.70, 0.81]
Sex	0.03	0.05	0.56	0.58	[−0.07, 0.13]
Age	<0.01	<0.01	1.70	0.09	[<−0.01, 0.01]
Education	0.02	0.02	0.84	0.40	[−0.02, 0.06]
Religiosity	−0.01	0.01	−1.25	0.21	[−0.04, 0.01]

Restrictive migration policy preferences were directly predicted by the hegemonic dimension of conservatism and negative attitudes toward migrants. As in the previous model with the palliative dimension of conservatism, negative attitudes toward migrants showed the strongest positive effect among all observed predictors. Together with covariates, the hegemonic dimension of conservatism and negative attitudes toward migrants explained 63% of the variance of restrictive migration policy preferences.

**Figure 3:**
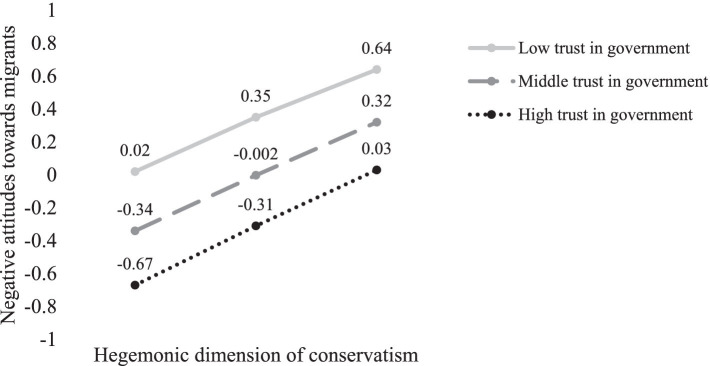
Conditional effects of the hegemonic dimension of conservatism on negative attitudes toward migrants at different values of trust in government. Figure shows unstandardized regression coefficients.

Conditional effects of the hegemonic dimension of conservatism on negative attitudes toward migrants at different values of trust in government are presented in Figure 3. The figure illustrates a very similar significant positive linear effect of the hegemonic dimension of conservatism on negative attitudes toward migrants at all levels of trust in government. In contrast to the previous model with the palliative dimension of conservatism, however, the 95% bootstrap confidence interval for the moderated mediation index included zero (*Index* = 0.02; *BootSE* = 0.03; *95% CI* [−0.04, 0.07]), indicating that the indirect effect of the hegemonic dimension of conservatism on restrictive migration policy preferences through negative attitudes toward migrants was not moderated by trust in government.

## Discussion

The present study investigated and disentangled associations of the palliative and hegemonic dimensions of conservatism with attitudes toward migrants and restrictive migration policy preferences. Although the evidence on their relationships abounds in the literature (Skitka et al., 2002; Inbar et al., 2009; Jost et al., 2009; Luguri et al., 2012; Hainmueller and Hopkins, 2014; Kugler et al., 2014; Brooks et al., 2016; Anderson and Ferguson, 2018; Cowling et al., 2019), the present study contributes to theory by showing that conservatism should not be conceived as a monolithic construct (Duckitt and Sibley, 2010, 2016). Not only may hegemonic and palliative dimensions of conservatism meet different psychological needs, they may also be associated with different attitudes toward migrants and migration policy preferences (Bertin et al., 2022; Górska et al., 2022). Furthermore, the study explored whether trust in government could mitigate negative attitudes toward migrants among people endorsing conservative beliefs. By disentangling motivations that drive the attraction to conservative views, the study indicates that trust in government could mitigate fear-induced negative attitudes toward migrants. However, the study also points to the limited ability of such trust to curb negative attitudes toward migrants motivated by deeply rooted radical or supremacist beliefs.

### Disentangling dimensions of conservatism and their consequences for attitudes toward migrants and restrictive migration policy preferences

Our results show that people high in the palliative dimension of conservatism, who are strongly attached to traditional norms and values, reported a greater sense of threat, fear, and fear-based xenophobia and showed more concern for the possible decay of norms caused by migrants. Unsurprisingly, those who are afraid of migrants and the damage that they may cause want to keep the source of their fear at a distance. Indeed, our mediation analysis suggests that restrictive migration policy preferences of those endorsing conservative views for palliative reasons are motivated indirectly by negative attitudes toward migrants. Interestingly, compared to the palliative dimension, the hegemonic dimension of conservatism showed weaker associations with fear-induced negative attitudes toward migrants. In other words, negative sentiments such as fear-based xenophobia and the sense of threat, although present, were not as strong as in the case of people who rely on conservative and traditional values to alleviate the sense of threat and uncertainty.

Generally, our results are in line with extant literature that shows that negative attitudes toward migrants have various sources but that one of the most prominent is fear and the sense of either a realistic or a symbolic threat posed by members of outgroups (Jost et al., 2003, 2017; Raijman and Semyonov, 2004). People experiencing a heightened sense of threat may seek consolation in traditional and conservative values as cornerstones of stability (Sinn and Hayes, 2018; De Cristofaro et al., 2019; Pazhoohi and Kingstone, 2021; Pless and Houtman, 2021). In this regard, our findings are consistent with the DPM model (Duckitt and Sibley, 2010, 2016). Our findings, however, also indicate that individuals with supremacist views did not experience feelings of threat and fear to the same extent as those high in the palliative dimension of conservatism. Nevertheless, even if migrants are not seen as being a severe threat to traditional values and norms, they are certainly not welcomed in the country either (Górska et al., 2022). In line with previous literature, our results show that the hegemonic dimension of conservatism is directly associated with preferences for restrictive migration policies (Ho et al., 2015; Anderson J., 2018; Anderson J. R., 2018; Cowling et al., 2019).

### Institutional trust as a shield against fear of migrants

Perhaps the most important and most promising finding of the study is that trust in government moderates the relationship between the palliative dimension of conservatism and attitudes toward migrants. In particular, as trust increased, the relationship between the palliative dimension of conservatism and negative attitudes weakened. This indicates that trust could serve as a protective factor against negative attitudes toward migrants for those conservatives who seek palliative relief. In line with extensive literature (Husfeldt, 2004, 2006; Halapuu et al., 2013; Economidou et al., 2017; McLaren, 2017; Serrano-Maillo, 2018; Jylhä et al., 2022), societal interventions focusing on increasing trust in government, as a direct proponent of migration policies, could help to lower negative attitudes toward migrants and subsequently increase the support for more inclusive migration policies.

In contrast, those who endorse conservatism motivated by hegemonic reasons and who do not experience heightened levels of fear and threat seem to be immune to the protective role of trust in government. Our results showed that the relationship between the hegemonic dimension of conservatism and attitudes toward migrants remained stable regardless of the level of trust in government. Therefore, for people scoring high in SDO and CN, interventions increasing trust may turn out to be futile — they feel less afraid of and threatened by migrants and, thus, do not need external reassurance provided by government. For them, the main driver of policy preferences may be rather a deep sense of superiority over and contempt for members of outgroups (Sherif, 1966). Regardless of the level of trust in government, people high in SDO and CN are prone to perceiving migrants to be inferior, having aversive xenophobic attitudes, and showing a preference for more restrictive migration policies.

Taken together, thus, our findings corroborate the view that there is a reason to disentangle motivational roots of conservative beliefs (Duckitt and Sibley, 2010, 2016). Our study points to the fact that trust in government is far from being a universal cure and can only be efficient when conservatism responds to the palliative needs to sooth the fear associated with migrants. People experiencing fear may feel more secure when they perceive their government to be a trustworthy guarantor of stability. Importantly, our measure of trust included questions on confidence in the government’s ability to manage the migration crisis efficiently. Therefore, it seems to be plausible that people who experience fear because of migrants may feel reassured by the belief that their government is competent in preventing threats from materialising. Consequently, this confidence may attenuate their negative attitudes toward migrants and enhance the support for more inclusive migration policies.

### Limitations and directions for future research

Despite our best efforts, the study is not free from limitations. Firstly, the study is cross-sectional and, thus, unable to grasp dynamics and long-term tendencies. A longitudinal and cross-country approach could be employed to trace whether changes in institutional trust are followed by changes in attitudes toward migrants and migration policy preferences. Moreover, it is possible that trust in government plays a stronger role once the government programme is congruent with personal beliefs or political attitudes; therefore, it would be recommended to observe possible changes in attitudes and the robustness of these relationships after elections (Kim, 2022).

Secondly, the study does not include some important aspects of conservatism. Specifically, we did not measure right-wing authoritarianism, which comprises both radical views and submissiveness to authorities and could bring fine-grained information on the relationships between conservatism and trust in government. Furthermore, our study focuses mainly on fear and threat and does not take into account other negative emotions that are likely to arise during contact with migrants or members of outgroups, such as contempt and revulsion (Sherif, 1966). Future studies could draw on the present findings and attempt to extend the perspective provided by our results.

Thirdly, it is important to point out that the study was conducted in Slovakia, which has a specific socio-political context in terms of attitudes toward migrants. In particular, Slovakia is one of the countries with rather limited experience with immigration, as few asylum applications are made there and even fewer are granted (Bozogáňová and Pethö, 2022). As Slovakia is not a traditional final destination for migrants, past European migration crises have not significantly affected the country’s cultural homogeneity. The lack of experience and contact with migrants places Slovaks at the top of the European rankings in terms of the levels of negative attitudes toward migrants and expected negative consequences of migration (Bozogáňová and Piterová, 2020). Future studies could focus on whether our results are generalizable to other countries that have positive attitudes toward migrants such as Sweden, Norway, Spain or Portugal.

## Conclusion

Although strengthening the trust that government manages the migration crisis with competence and having best interests of citizens in mind may seem to be a promising method for mitigating negative attitudes toward migrants and enhance preferences for more inclusive migration policies, it can also be a challenging strategy. In many countries, institutional trust is waning or stagnating and there is no simple method with which to increase it, as it is a context-sensitive phenomenon related to the experience of existential threats and the sense of insecurity (Perry, 2021). Clearly, when governments and institutions are subjectively perceived to be knowledgeable about migration and are trusted by the citizens, this could mitigate the sense of threat and, thus, attenuate negative or hostile attitudes toward migrants by signalling that they efficiently manage the alleged (or real) risks associated with migrants. In other words, when people believe they are in good hands of competent politicians they may feel reassured that whatever the migration policies and measures are introduced they are sufficient to tackle the risks and protect citizens from either real or symbolic threats posed by the migrants. However, reducing the sense of threat is not always consistent with political interests. Politicians often skilfully play the card of threats – whether actual or imagined – including those allegedly caused by migrants, to consolidate support for their parties and political programmes (including more restrictive migration policies). By embroidering this threat, politicians may use the fear of migrants to portray themselves as sole guardians and beacons of traditional values to make more political capital and seize power. In other words, not only could trust be used as a tool with which to combat the public’s fear, feelings of threat could also be exploited to increase support for certain politicians as those who could be trusted to solve the burning issue efficiently.

## Data availability statement

The original contributions presented in the study are included in the article/supplementary materials, further inquiries can be directed to the corresponding author.

## Ethics statement

The studies involving humans were approved by Ethical Committee of the Centre of Social and Psychological Sciences of the Slovak Academy of Sciences. The studies were conducted in accordance with the local legislation and institutional requirements. The participants provided their written informed consent to participate in this study.

## Author contributions

MG: Conceptualization, Data curation, Formal analysis, Funding acquisition, Investigation, Methodology, Project administration, Resources, Visualization, Writing – original draft. MA: Conceptualization, Investigation, Methodology, Project administration, Supervision, Writing – original draft. JT: Writing – original draft.

## Footnotes

^1^https://osf.io/6skz9/?view_only=f39b92b086bc4bcc88a86ed565484d7d
